# Do Neutrophils Play a Role in Establishing Liver Abscesses and Distant Metastases Caused by *Klebsiella pneumoniae*?

**DOI:** 10.1371/journal.pone.0015005

**Published:** 2010-11-30

**Authors:** Jung-Chung Lin, Feng-Yee Chang, Chang-Phone Fung, Kuo-Ming Yeh, Chiung-Tong Chen, Yu-Kuo Tsai, L. Kristopher Siu

**Affiliations:** 1 Division of Infectious Diseases and Tropical Medicine, Department of Internal Medicine, Tri-Service General Hospital, National Defense Medical Center, Taipei, Taiwan; 2 Institute of Clinical Medicine, School of Medicine, National Yang-Ming University, Taipei, Taiwan; 3 Division of Biotechnology and Pharmaceutical Research, National Health Research Institutes, Taipei, Taiwan; 4 Division of Infectious Diseases, National Health Research Institutes, Taipei, Taiwan; Institut Pasteur, France

## Abstract

Serotype K1 *Klebsiella pneumoniae* is a major cause of liver abscesses and endophthalmitis. This study was designed to identify the role of neutrophils in the development of distant metastatic complications that were caused by serotype K1 *K. pneumoniae*. An *in vitro* cellular model was used to assess serum resistance and neutrophil-mediated killing. BALB/c mice were injected with neutrophils containing phagocytosed *K. pneumoniae*. Serotype K1 *K. pneumoniae* was significantly more resistant to serum killing, neutrophil-mediated phagocytosis and intra-cellular killing than non-K1 isolates (p<0.01). Electron microscopic examination had similar findings as in the bioassay findings. Intraperitoneal injection of neutrophils containing phagocytosed serotype K1 *K. pneumoniae* led to abscess formation in multiple sites including the subcutaneous tissue, lung, and liver, whereas no abscess formation was observed in mice injected with non-K1 isolates. The resistance of serotype K1 *K. pneumoniae* to complement- and neutrophil-mediated intracellular killing results in the dissemination of *K. pneumoniae* via the bloodstream. Escape from neutrophil intracellular killing may contribute to the dissemination and establishment of distant metastases. Thus, neutrophils play a role as a vehicle for helping *K. pneumoniae* and contributing to the establishment of liver abscess and distant metastatic complications.

## Introduction

*Klebsiella pneumoniae* is the major pathogen associated with pyogenic liver abscesses [1]–[5]. Most patients with *K. pneumoniae* liver abscess develop bacteremia and septic metastatic complications including endophthalmitis, meningitis, brain and lung abscesses, and necrotizing fasciitis [5]–[11]. In Europe and North America, the frequencies of *K. pneumoniae* capsular serotypes K2 and K21 are greater than those of other serotypes [12]. Serotype K1 is the most common serotype of *K. pneumoniae* isolated from the blood, urine, respiratory tract, and pus (especially from liver abscesses) in Taiwan [13]. However, serotype K1/K2 is the most common cause of liver abscesses [2]. Septic endogenous endophthalmitis, which occurs with a frequency of 3.3–10%, indicates a poor prognosis. Furthermore, 60–93% of patients with liver abscesses and distant metastasis of endophthalmitis were reported to be diabetic [1], [2], [7], [11], [13]–[17].

The association of *K. pneumoniae* with liver abscesses reflects the substantial pathogenic arsenal of the bacterium (including capsular K antigens, serum resistance, the possession of fimbriae and lipopolysaccharide, and the expression of siderophores) [2], [18]. Less well known, however, is the importance of serotype K1 in the pathogenesis of *K. pneumoniae* liver abscess with distant metastasis. Bacterial dissemination through the bloodstream is generally believed to promote distant metastasis. However, innate defense mechanisms involving mediators of bacterial cell killing such as polymorphonuclear neutrophils (PMNs) and serum complement are involved in preventing infection and dissemination. Although PMNs are a key anti-infection factor in host defense, they can also cause tissue damage. Previous studies have focused on the molecular mechanisms governing phagocytosis and bacterial killing [19]. The role of these effector functions is not clear when the phagocytosed bacteria are resistant to intracellular killing and when circulating neutrophils are the cause of distant infection. The mechanisms used by *K. pneumoniae* to colonize, invade, infect, disseminate, and disrupt the host defense are diverse. Each phase of the process involves the interaction of a variety of bacterial and host factors that cause disease. Although neutrophils participate in the first line of defense against bacterial disease, they may no longer play a defensive role when specific bacterial factors are involved. In the present study, we postulated that circulating neutrophils containing serotype K1 *K. pneumoniae* which are resistant to intracellular killing serve as a vector for disseminating the bacteria to the liver and distant metastatic sites.

## Results

### Serum bactericidal assay

A total of 37 serotype K1 *K. pneumoniae* isolates were selected for use in a serum bactericidal assay. Of these, 20 isolates originated from liver abscess. Isolates of serotypes other than K1, 17 isolates, were clinical isolates from sources such as blood, stool and urine. All isolates were incubated with pooled human sera for various time intervals. Serotype K1 *K. pneumoniae* isolates from liver abscesses were significantly more serum resistant than isolates from non-liver abscesses (*p* = 0.01). In contrast, the serum resistance of non-K1/K2 strains (one isolate each of K3, K6, K15, K16, K17, K20, K21, K22, K29, K31, K36, K38, and K54, and two isolates each of K55 and K28) from liver abscesses was not significantly different from isolates of the same strains from non-liver abscesses in terms of serum resistance (*p* = 0.12). Generally, the serum resistance of K1 isolates was higher than that of non-K1/K2 isolates (*p* = 0.0019) (Table 1).

**Table 1:** Distribution of serum-sensitive and serum-resistant serotypes of *K. pneumoniae* selected for this study.

Serotypes	No. of isolates		*p* value
	Sensitivity (Grade 1–4)	Resistance (Grade 5–6)	
K1 (n = 20)	4	16	
Non-K1/K2 (n = 17)*	12	5	
K1 *vs.* non-K1/K2			0.0019

*The 17 strains of non-K1/K2 contained one isolate each of K3, K6, K15, K16, K17, K20, K21, K22, K29, K31, K36, K38, and K54 and two isolates each of K55 and K28. The grading system used to assess serum resistance was described by Podschun et al. (1991).

**Figure 1:**
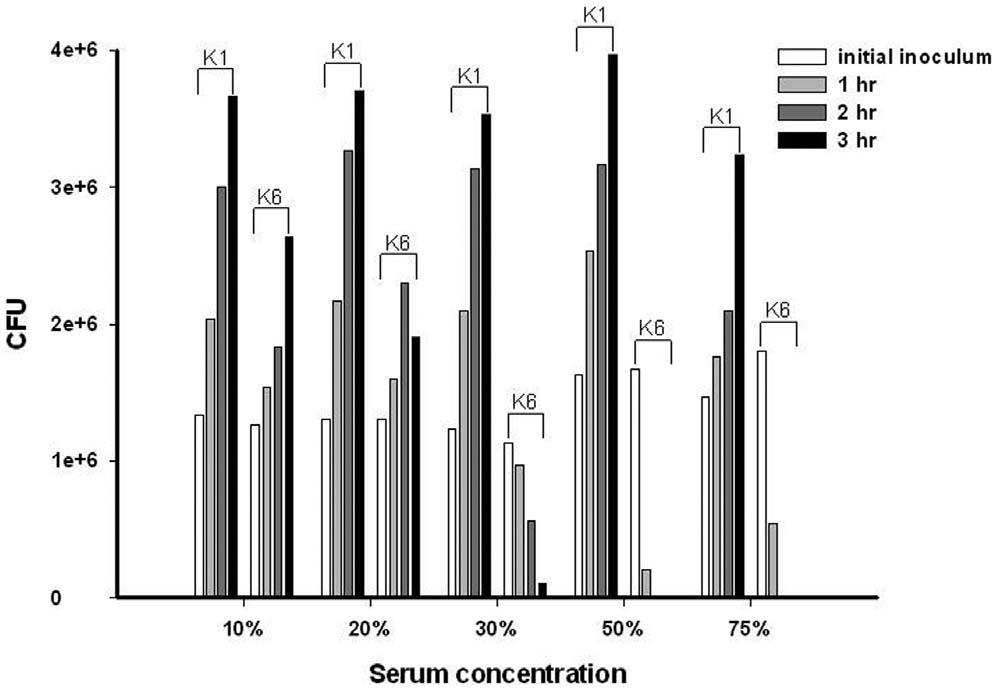
The effect of serum dilution on the serum killing of *K. pneumoniae* serotypes K1 and K6. A high concentration of serum inhibited the growth of K6 but not K1. Each experiment was repeated in triplicate. The bacteria counts are expressed by cell forming unit (CFU).

To confirm the strength and specificity of the serum effect, a serum bactericidal assay was performed with different concentrations of serum. Concentrations ranging from 10 to 75% serum were incubated with serotypes K1 and K6 *K. pneumoniae* (ATCC700603), respectively. Grade 6 level serum resistance was evident for K1 over the serum concentration range 10–75% (Fig. 1). For serotype K6, grade 5 or 6 level serum resistance was evident at serum concentrations at a range of 10–20%, but no serum resistance was evident at a range of 30–75% (Fig. 1). High concentrations of serum inhibited the growth of K6, but not K1.

### Viability of serotype K1 and K6 *K. pneumoniae* following phagocytosis by neutrophils

**Figure 2:**
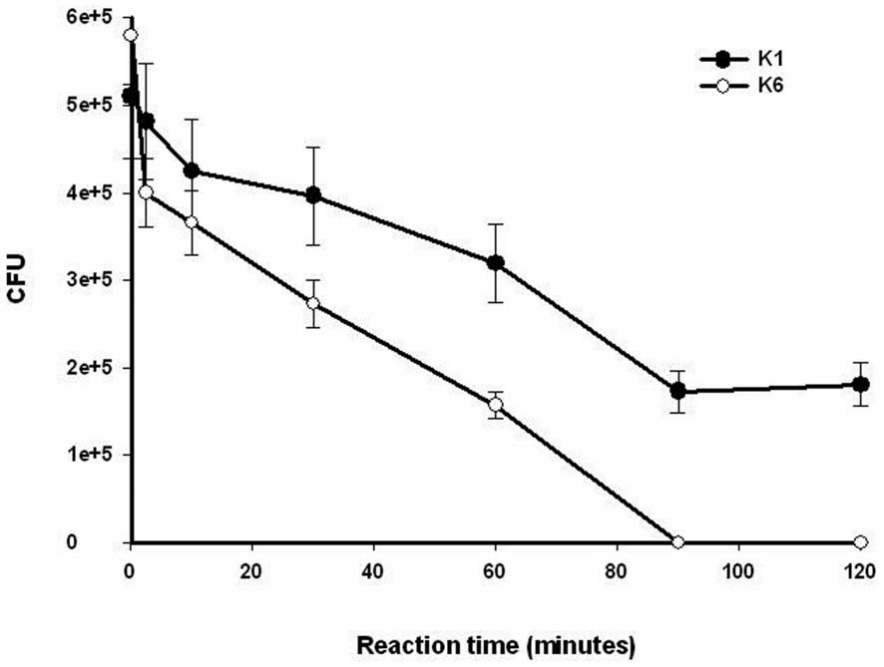
The rate at which neutrophils from 11 healthy volunteers killed serotypes K1 and K6 *K. pneumoniae*. The percentage of killing is expressed as the mean ± SD and calculated as the total number of bacteria killed divided by the number of viable bacteria in the initial inoculum. K1 vs. K6: *p*<0.01 (n = 3 for each).

The decline in viable bacterial counts in the neutrophil phagocytosis assay was slower for serotype K1 than serotype K6 (Fig. 2). The viable counts of serotype K1 stabilized and increased modestly after 90 minutes of phagocytosis, whereas the viable counts of serotype K6 declined sharply to below the limit of detection after 90 minutes of phagocytosis (Fig. 2). The difference in the counts of viable intracellular bacteria between phagocytosed K1 and K6 was statistically significant (*p*<0.01).

### Electron microscopic evaluation of intracellular killing

**Figure 3:**
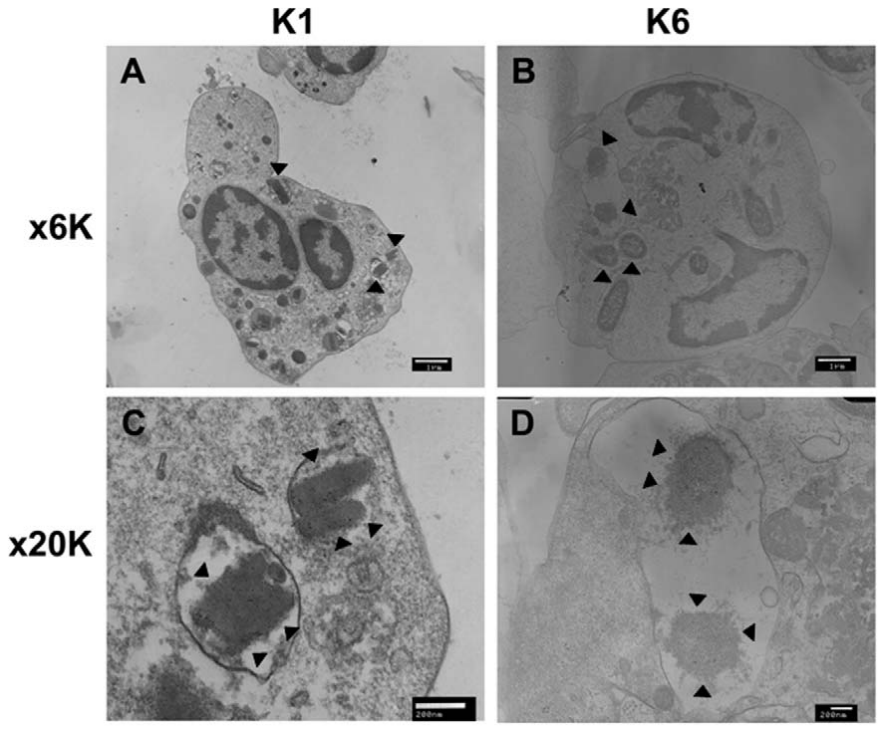
Electron microscopic examination of phagocytosed serotypes K1 and K6 *K. pneumoniae*. Vacuolated bacteria are evident. The cell wall structure of serotype K1 appears intact, and cell division patterns are visible despite digestion by neutrophils (A & C). In contrast, lysis and the loss of cell wall structure of serotype K6 is seen in the vacuoles of neutrophils (B & D). (Bar = 1 µm, cells magnified ×6K; bar = 200 nm, cells magnified ×20K). Bacteria are pointed by arrow.

Following phagocytosis, K1 and K6 bacteria were evident within vacuoles. While changes in K1 cell wall morphology were minimal (Fig. 3A), K6 cell walls were completely disrupted, resulting in lysis (Fig. 3B).

### Inoculation of neutrophil-phagocytosed serotype K1 and K6 *K. pneumoniae*

**Figure 4:**
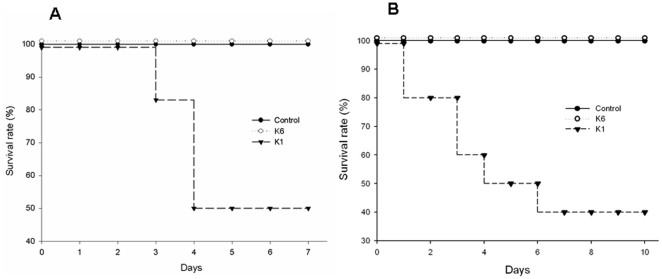
Survival rate of BALB/c mice (n = 12 per group) following intraperitoneal injection of neutrophils containing phagocytosed serotypes K1 and K6 *K. pneumoniae*. The number of viable intracellular bacteria were 4×10^3^ and 5×10^5^ for serotypes K1 and K6. Survival was observed for 10 days. Each experiment was repeated twice. (A) Human neutrophils. (B) BALB/c neutrophils. (*p*<0.001, for the serotype K1 group *vs*. the K6 and control groups in human and mouse experiments.)

Survival after infection with neutrophil-phagocytosed serotypes K1 and K6 *K. pneumoniae* was assessed using a murine model. The number of viable bacteria phagocytosed by human neutrophils averaged 4×10^3^ and 5×10^5^ for serotypes K1 and K6, respectively. Mouse mortality was significantly higher (*p*<0.01) in mice receiving human neutrophils containing phagocytosed K1 than those receiving human neutrophils containing phagocytosed K6 (Fig. 4A). No mortality was observed in mice that received neutrophils containing K6 or bacteria-free neutrophils. The number of viable bacteria phagocytosed by mouse neutrophils averaged 7×10^3^ and 6×10^5^ for serotypes K1 and K6, respectively. Whether the source of the neutrophils containing serotype K1 or K6 *K. pneumoniae* was mouse or human, the results of their administration were similar (Fig. 4B), indicating that mortality in mice was not caused by the immune incompatibility of human neutrophils.

### Gross and molecular examination of abscess formation after intraperitoneal inoculation of neutrophils

**Figure 5:**
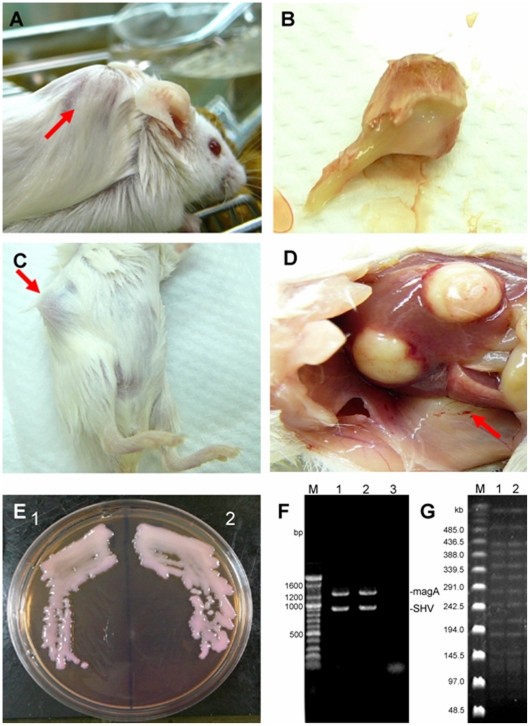
Gross abscess appearance in mice injected with neutrophils containing phagocytosed serotype K1 *K. pneumoniae*. A: Neck. B: Surgical removal of the abscess site. C: Abdomen. D: Abscess formation in liver and subcutaneous areas. E: (1) Morphological features of injected serotype K1 *K. pneumoniae* and (2) culture of pus discharge from the abscess site. F: Detection of the serotype K1 specific *magA* gene and intrinsic *bla*_SHV_ gene from (1) the parental strain and (2) bacteria isolated from the abscess site. G: PFGE molecular typing of (1) the parental strain and (2) bacteria isolated from the abscess site. Sites of abscess formation are indicated by red arrows.

Abscess formation was assessed by gross examination after intraperitoneal (IP) inoculation of serotype K1 *K. pneumoniae*. All mice died after the inoculation of 4.75×10^4^ colony-forming units (cfu) of phagocytosed serotype K1, whereas no mice died following inoculation with a similar dose of phagocytosed serotype K6 (Table 2). Abscesses formed on the necks (Fig. 5A and 5B) and abdomens of mice that received serotype K1 (range, 2.56×10^2^ to 4.83×10^3^; Fig. 5C and 5D). Abscesses on the neck formed after the administration of approximately 10^3^ cfu, but not following a dose of 10^2^ cfu. The percentage of mice developing abdominal abscesses did not increase with increasing K1 inoculum (10^2^ to 10^3^ cfu; Table 2).

**Table 2:** Mortality and abscess formation after intraperitoneal (IP) injection of neutrophil-phagocytosed serotype K1 and K6 *K. pneumoniae.*

Serotype (no. of mice)	Dose (cfu)	Mortality (%)	Abscess formation	
			Neck (%)	Abdomen (%)
K1(10)	4.75×10^4^	10 (100)	0	0
K6 (10)		0	0	0
K1 (119)*				
Range	1.42–4.83×10^3^	59 (49.58)	6 (5.04)	8 (6.72)
K1 (50)†				
Range	2.56–7.90×10^2^	18 (36.00)	0	8 (16.00)

*10 groups of mice: 8 groups (each n = 10), 1 group (n = 19), and 1 group (n = 20) were injected IP with 10^3^ cfu of neutrophil-phagocytosed serotype K1.

† 5 groups (each n = 10) were injected IP with 10^2^ cfu of neutrophil-phagocytosed serotype K1.

The isolate recovered from pus discharge and the inoculated serotype K1 isolate were morphologically identical, and both had *magA* (Fig. 5E and 5F). Molecular typing by PFGE showed that the strain isolated from the liver was identical to the K1 strain of *K. pneumoniae* that had been parenterally administered (Fig. 5G).

### Pathological features at liver, spleen, lung, kidney, and metastatic infection sites

**Figure 6:**
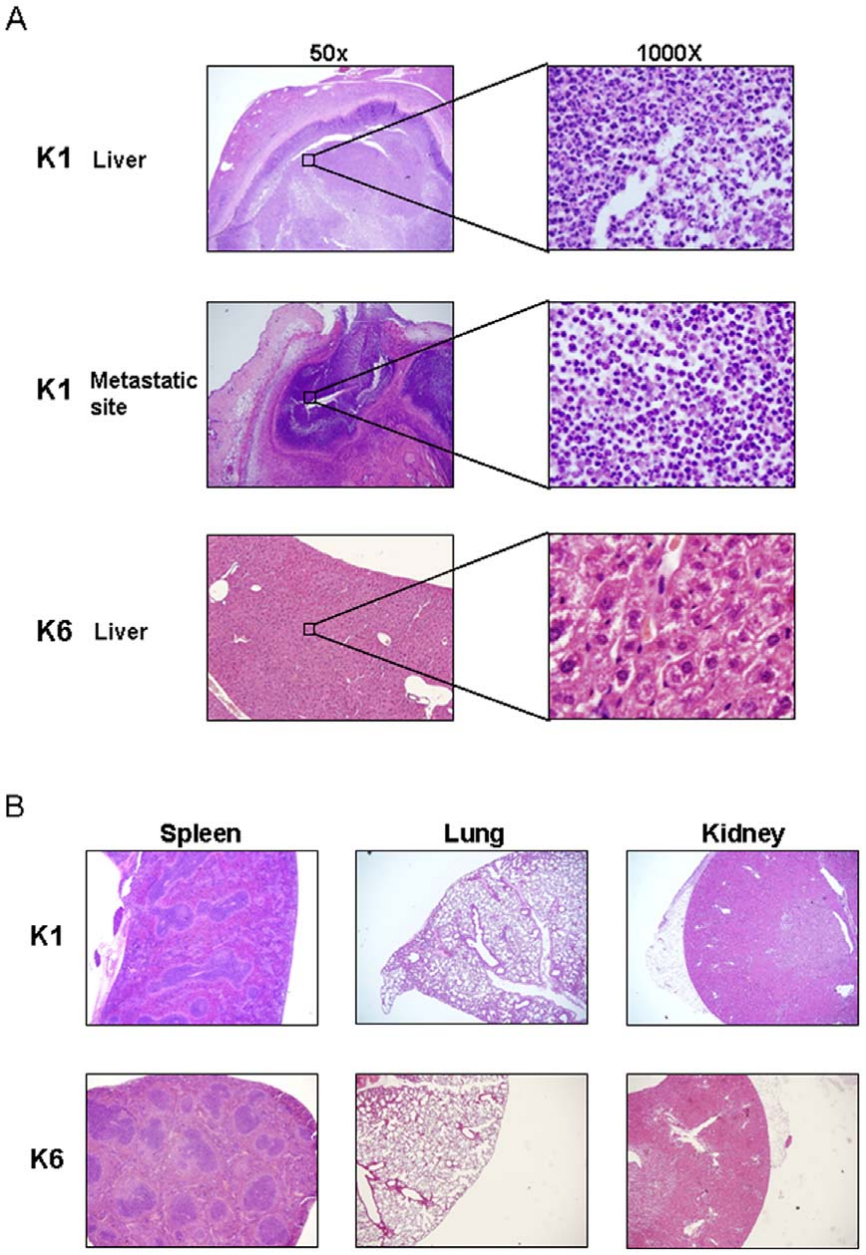
Pathologic features of abscesses due to serotypes K1 and K6 *K. pneumoniae* in different mouse tissues. (A). Necrosis can be seen in the liver and at metastatic infection sites in mice injected with K1 *K. pneumoniae*. No inflammation was seen in the liver of mice injected with serotype K6. (B). Inflammation was not found in the lungs, spleen, or kidneys of mice injected with serotypes K1 and K6.

Necrosis was identified in tissue sections from sites of metastatic infection as well as the liver, but not in the lung, spleen, or kidney of mice injected with serotype K1 (Fig. 6A and 6B). Inflammation was not evident in liver, spleen, lung, or kidney samples from mice receiving serotype K6. Numerous PMN and monocytes had infiltrated liver and subcutaneous tissue (a metastatic infection site), indicating severe inflammation. *K. pneumoniae* were identified in liver and subcutaneous tissues sections (Fig. 6A).

## Discussion

In this study, serotype K1 was found to be more resistant to killing by both serum killing (*p*<0.01) (Fig. 1) and intracellular killing after neutrophil phagocytosis (*p*<0.01) (Fig. 2) than non-K1 serotypes in cellular and murine models. Electron microscopy agreed with the finding of the bioassay (Fig. 3). Furthermore, cells that remained viable for extended periods (i.e., phagocytosed K1 cells), but not phagocytosed K6 cells, were linked to lethal septicemia (Fig. 4A and 4B) and abscess formation in mice (Figs. 5, 6).

Clinical infection by serotypes K2, K7, and K21 has been linked to the resistance of the polysaccharide capsule of *K. pneumoniae* to phagocytosis and serum killing [20]–[22]. Previously, we observed that serotype K1 is the main serotype in *K. pneumoniae*-mediated liver abscess and is associated with the development of endophthalmitis and meningitis, particularly in diabetic patients [2]. Therefore, it was of interest to understand how K1 virulence factors, particularly the capsule, contribute to the pathogenesis of *K. pneumoniae*-mediated liver abscess and other complications of infection. Colonization of the gut by *K. pneumoniae* is believed to be the first step in *K. pneumoniae* liver abscess development. The intestine communicates with the liver via the hepatic portal system, and a transient bacteremia of bowel flora (such as *Escherichia coli*) is not uncommon [23], although it remains to be determined how this bacterium passes through the gut epithelial barrier into the bloodstream. Neutrophils and serum complement are key factors in preventing bacterial multiplication leading to further dissemination. However, phagocytosis of *K. pneumoniae* is also part of the infectious process, as neutrophils carrying phagocytosed *K. pneumoniae* may circulate in the bloodstream and transport K1 bacteria to distant sites causing abscess formation. The studies presented herein suggest that neutrophils containing phagocytosed K1 were lysed by the immune system shortly after their injection into mice, releasing free K1 bacteria that then disseminated through the bloodstream. However, we previously showed that injection of as few as <10 cfu of free K1 could induce septic shock, leading to multiorgan failure and death in all mice within four days [24]. The chronic development of abscesses at the liver or other sites was not observed. In the present model using neutrophils containing phagocytosed K1 bacteria, at least ≥10^2^ cfu were required for the development of liver and neck abscesses. Abscess formation became grossly apparent around 18–21 days, indicating a chronic infection. If neutrophils containing phagocytosed K1 were rapidly lysed after injection, they should have caused mortality even sooner than free K1 bacteria because the inoculum released from neutrophils contained a greater number of bacteria. Thus, immigrating neutrophils play a role in killing bacteria trapped in the liver [25].

Bacteremia frequently occurs in *K. pneumoniae* liver abscesses with septic endophthalmitis via portal entry and perhaps via the bloodstream [1], [5], [7], [11]. This study demonstrated the ability of bacteria to escape serum lysis and neutrophil killing during their passage through the circulatory system. Our results indicate that serum resistance and intracellular survival within neutrophils contribute to the pathogenesis of this disease. Previous studies have shown that bacterial pathogens such as *Mycobacterium tuberculosis* and *Salmonella typhimurium* persist for years within phagocytic cells such as macrophages and neutrophils, and within lung granulomas and mesenteric lymph nodes of humans [26], [27]. These pathogens will sometimes reactivate, causing an acute infection. During instances of typhoid fever, *Salmonella*-infected phagocytes gain access to the lymphatic system and bloodstream, facilitating bacterial spread to the liver. Whether *Kelbsiella*-infected neutrophils gain access through a similar means, leading to liver abscess and distant metastasis, remains to be shown. Further studies are needed to confirm this hypothesis.

Serotypes K1 and K2 are associated with complicated endophthalmitis, particularly in cases of diabetes mellitus, which occurs in 93% of afflicted patients [2]. A Diabetes-related reduction in the efficiency of phagocytosis, chemotaxis, and intracellular killing has been reported [28]–[32]. Although diabetes mellitus contributes to the pathogenesis of *K. pneumoniae*-induced liver abscess complicated with endophthalmitis [1], [9], [10], metastatic infection sites do not arise in all cases of liver abscess due to serotype K1/K2 bacteria. Whether septic endophthalmitis in patients without diabetes mellitus is due to differences in the virulence of serotype K1 bacteria remain to be elucidated. In the present *in vivo* experiments, the survival of serotype K1 *K. pneumoniae* within neutrophils and escape from serum-mediated killing contributed to the development of septic infections. One intriguing finding is that 12–16% of mice developed metastatic infections. These results are quite similar to the previous surveillance of liver abscesses in which approximately 10% of patients developed distant metastatic foci such as endophthalmitis or meningitis [2]. Metastatic infections in the neck were only found after the injection of a relatively high (1.42–4.83×10^3^ cfu), but not a low (2.56–7.90×10^2^ cfu) dose of phagocytosed K1. Furthermore, increasing the number of cells containing phagocytosed K1 bacteria increased mortality but not the percentage of mice developing distant metastases. These results reflect the contribution made by increasing numbers of phagocytosed K1 bacteria in both dissemination of the bacteria to distant sites and the severity of infection. In conclusion, neutrophils containing phagocytosed serotype K1 bacteria may contribute to the establishment of liver abscesses and distant metastasis in disease caused by hepatovirulent *K. pneumoniae.*

## Materials and Methods

### Bacterial strains

Thirty-seven strains of serotype K1 and non-K1/K2 *K. pneumoniae* were isolated from patients with liver abscesses complicated with endophthalmitis and from patients with non-liver abscesses. The study focused on serotype K1 because serotypes K1/K2 are linked to the development of liver abscess [2]. The capsular serotype was confirmed by a capsular swelling technique and countercurrent immunoelectrophoresis [13]. All strains were kept frozen at –80°C in beads containing a cryopreservative. Suspensions were established by an overnight incubation at 37°C in brain heart infusion broth prior to experiments. The concentration and viability of the bacteria were determined by quantitative plate counting using established techniques.

### Mice

Pathogen-free, 6–8 week-old, male BALB/c mice weighing 20–25 g were obtained from the National Laboratory Animal Center (Taipei, Taiwan). The protocols for all experiments involving mice were approved by the Committee on Institutional Animal Care and Use, Tri-Service General Hospital and National Defense Medical Hospital (IACUC-05-166).

### Preparation of normal human and BALB/c sera

Normal human serum was obtained from ten healthy volunteers, who had provided informed written consent. Sixty milliliters of freshly drawn heparin-free blood was clotted at room temperature. BALB/c serum was prepared following heart puncture after inhalation of carbon monoxide. The clotted blood was centrifuged at 1000× *g* for 20 min at 20°C. The sera were pooled, and aliquots were stored at –70°C until use.

### Isolation of human and BALB/c mouse neutrophils

Isolation of neutrophils from healthy volunteers met ethically approved guidelines [33], [34]. Healthy volunteers were screened after giving informed written consent [34]. Neutrophils from BALB/c mice were isolated from the peritoneum as previously described [33]. The cell concentration was adjusted to 1×10^7^ cells/mL. The viability of the isolated neutrophils exceeded 95%, as determined by Trypan blue exclusion. All procedures involving animals and humans were approved by the Tri-Service General Hospital Institutional Review Board and the Committee on Institutional Animal Care, Use, and Ethics, National Defense Medical Center (TSGHIRB 095-05-0054 and IACUC-05-166).

### *In vitro* serum bactericidal assay

Serum bactericidal activity was measured as described previously with slight modifications [22], [35]. Bacteria grown in nutrient broth were collected during the early logarithmic phase. The viable bacterial concentration was adjusted to 1×10^6^ cfu/mL. Twenty-five microliters of bacteria was added to 75 µL of pooled human sera in a 10×75 mm Falcon polypropylene tube (BD Biosciences, Franklin Lakes, New Jersey). Tubes were agitated for 0, 60, 120, or 180 min. To determine the number of viable bacteria after exposure to serum, an aliquot of each bacterial suspension was removed at the designated time point, diluted 10-fold by the addition of Mueller-Hinton broth, plated on Mueller-Hinton agar, and assayed as described below. Results are expressed as percentage of the inoculum, and responses in terms of viable counts were graded from 1 to 6 as described previously [22], [35]. Grade 1 represented viable counts that were <10% of the inoculum after 1 and 2 h, and <0.1% after 3 h. Grade 2 represented viable counts that were 10–100% of the inoculum after 1 h and <10% after 3 h. Grade 3 represented viable counts that exceeded those of the inoculum after 1 h, but were <100% after 2 and 3 h. Grade 4 represented viable counts that were >100% of the inoculum after both 1 and 2 h, but <100% after 3 h. Grade 5 represented viable counts that were >100% of the inoculum after 1, 2, and 3 h but that decreased during the third hour. Finally, grade 6 represented viable counts that exceeded those of the inoculum after 1, 2, and 3 h and that increased throughout this time period. Each strain was tested at least three times. A strain was considered serum resistant or serum sensitive if the grading was the same in all experiments. Each isolate was classified as highly sensitive (grades 1 or 2), intermediately sensitive (grades 3 or 4), or resistant (grades 5 or 6).

### *In vitro* neutrophil bactericidal assay

A neutrophil bactericidal assay was performed as described previously, with slight modifications [36], [37]. Serum was thawed immediately prior to use and was kept on ice until assayed. Neutrophils (1×10^6^), 10% v/v pooled normal human serum, and viable *K. pneumoniae* (4×10^7^) were added to 10×75 mm polypropylene tubes (BD Biosciences). The capped tubes were incubated in a shaking water bath at 37°C with continuous agitation for 0, 2.5, 10, 30, 60, 90, or 120 min. Samples were removed at each timepoint and placed in an ice bath. Prior to counting bacteria that survived neutrophil phagocytosis, gentamicin (100 µg/ml) was used to kill extracellular and adherent bacteria and to ensure that no extracellular bacteria were counted as intracellular bacteria. Supernatants of culture medium were removed after incubation with gentamicin for 30 minutes and spread onto an agar plate to confirm that no viable bacteria were present before counting. After confirming that no viable extracellular bacteria or adherent bacteria were present, neutrophils were lysed with a hypotonic (sterilized double distilled water) solution for 5 min. Complete lysis of neutrophils was confirmed by flow cytometry. Adjusted zero was applied in the first tube, followed by the immediate addition of gentamicin and a 30 minute-incubation. Bacterial viability was determined as described above, and the reduction in viable numbers was used to determine the number of bacteria killed. Results are expressed as the percentage of bacteria killed over time: 100%× (1- [number of viable bacteria after adding neutrophils/number of viable bacteria before adding neutrophils]).

### Evaluation of intracellular killing by electron microscopy

Purified neutrophils were mixed with opsonized bacteria for 30 min under the conditions described above for the neutrophil killing assay. Extracellular undigested bacteria and adherent bacteria were killed by gentamicin (100 µg/ml, 37°C, 30 min). Cells were fixed as previously detailed [37], dehydrated in a graded ethanol series, and embedded in Eponate-12 (Ted Pella, Redding, California). Ultra-thin sections were stained with uranyl acetate and lead citrate (Electron Microscopy Sciences, Fort Washington, Pennsylvania) and examined using a JEOL 1230 transmission electron microscope (JEOL, Tokyo, Japan) as described previously [38].

### Survival of *K. pneumoniae* after neutrophil-mediated phagocytosis

To confirm whether *K. pneumoniae* can survive after phagocytosis and contribute to infection, purified neutrophils (1×10^6^ cells/mL) from normal healthy subjects or BALB/c mice were incubated with opsonized and viable K1 or K6 *K. pneumoniae* (4×10^7^ cells/mL), respectively, for 30 minutes and then treated with gentamicin (100 µg/ml, 37°C, 30 min). The number of phagocytosed bacteria was determined by hypotonic lysis and the plating of serially diluted preparations. Neutrophils (1×10^5^ cells/mL) containing viable intracellular bacteria were injected intraperitoneally (IP) into BALB/c mice. An equivalent number of similarly injected neutrophils without intracellular bacteria served as controls. Mortality was assessed for up to 10 days. Each experiment was repeated twice.

### Murine sepsis model

To replicate murine IP-derived sepsis experimentally, BALB/c mice were injected with varying numbers of viable forms of K1 or K6 serotypes of *K. pneumoniae*. The mice were scored for their gross appearance every 24 h for 21 days, after which they were sacrificed. The liver, lungs, and spleen were removed aseptically, fixed with 10% (v/v) formalin and embedded in paraffin. Histological sections were stained using hematoxylin and eosin stain (H&E stain) and observed by light microscopy.

### Pulsed-field gel electrophoresis (PFGE) analysis

Total DNA was prepared and PFGE was performed as described previously [39]. The restriction enzyme *Xba*I (New England Biolabs, Beverly, MA, USA) was used at the manufacturer's suggested temperature. Restriction fragments were separated by PFGE in 1% agarose gel (Bio-Rad, Hercules, CA, USA) in 0.5×TBE buffer (45 mM Tris, 45 mM boric acid, 1.0 mM EDTA, pH 8.0) for 22 h at 200 V at a temperature of 14°C, with ramp times of 2 to 40 s using the Bio-Rad CHEF MAPPER apparatus (Bio-Rad Laboratories, Richmond, CA, USA). Gels were then stained with ethidium bromide and photographed under ultraviolet light. The resulting genomic DNA profiles, or “fingerprints”, were interpreted according to established guidelines [40].

### Statistical analyses

Multivariate analyses were performed to compare differences in the adjusted serum resistance rates and neutrophil killing rates between K1 and non-K1/K2 serotypes. If significant results were found, these variables were examined by univariate analyses. Data are expressed as least squares mean (LSMEAN) ± standard error of the mean (SEM). Statistical analyses of survival curves were conducted using the log rank test. All statistical tests were two-sided, and *p*-values <0.05 were considered to be statistically significant.
